# Supplementary material for: D-index and invasive fungal infections (IFIs) in adult acute myeloid leukemia (AML) patients with the first episode of febrile neutropenia
Source: PLoS One. 2023 May 22;18(5):e0286089. doi: 10.1371/journal.pone.0286089 (PMC10202296; doi:10.1371/journal.pone.0286089)
Supplement: S1 Checklist — (DOCX) [file pone.0286089.s002.docx]

STROBE Statement—checklist of items that should be included in reports of observational studies

|  | Item No. | Recommendation | Page  No. | Relevant text from manuscript |
| --- | --- | --- | --- | --- |
| **Title and abstract** | 1 | (*a*) Indicate the study’s design with a commonly used term in the title or the abstract | 2 | A retrospective study... |
|  |  | (*b*) Provide in the abstract an informative and balanced summary of what was done and what was found | 2 | The D-index and c-D-index were helpful indicators for defining the risk of IFIs in AML patients with febrile neutropenia. |
| Introduction | | | |  |
| Background/rationale | 2 | Explain the scientific background and rationale for the investigation being reported | 3-4 | As line 57-80 |
| Objectives | 3 | State specific objectives, including any prespecified hypotheses | 4 | Therefore, this study aimed to investigate the impacts of D-index performance on IFIs and other infectious complications in adult AML patients... |
| Methods | | | |  |
| Study design | 4 | Present key elements of study design early in the paper | 4 | A retrospective cohort... |
| Setting | 5 | Describe the setting, locations, and relevant dates, including periods of recruitment, exposure, follow-up, and data collection | 4 | ...between January 2014 to December 2020 at Chiang Mai University Hospital, Thailand. All clinical characteristics were collected by the retrospective chart review with de-identified patients' data... |
| Participants | 6 | (*a*) *Cohort study*—Give the eligibility criteria, and the sources and methods of selection of participants. Describe methods of follow-up  *Case-control study*—Give the eligibility criteria, and the sources and methods of case ascertainment and control selection. Give the rationale for the choice of cases and controls  *Cross-sectional study*—Give the eligibility criteria, and the sources and methods of selection of participants | 4-5 | (Line 89-117) Inclusion criteria included newly diagnosed de novo AML patients aged 18 years or above... |
|  |  | (*b*) *Cohort study*—For matched studies, give matching criteria and number of exposed and unexposed  *Case-control study*—For matched studies, give matching criteria and the number of controls per case |  |  |
| Variables | 7 | Clearly define all outcomes, exposures, predictors, potential confounders, and effect modifiers. Give diagnostic criteria, if applicable | 5-6 | Line 105-108: AML patients who developed IFIs were classified as possible, probable, or proven according to the revised criteria of invasive fungal disease from the European Organization for Research and Treatment of Cancer and the Mycoses Study Group Education and Research Consortium (EORTC/MSG).  Line 119-134: D-index and cumulative D-index (c-D-index) calculation ..... |
| Data sources/ measurement | 8* | For each variable of interest, give sources of data and details of methods of assessment (measurement). Describe comparability of assessment methods if there is more than one group | 4  7 | All clinical characteristics were collected by the retrospective chart review.  The descriptive statistics were described as mean ± standard deviation (SD), median with interquartile range (IQR), or percentage values as appropriate. Dichotomous variables were compared using the Chi-square or Fisher's exact test. The test of normality was assessed by the Shapiro-Wilk test. Comparison of continuous variables between IFIs and no-IFIs group was analyzed by Student's *t*-test (for parametric analysis) or Mann-Whitney U test (for non-parametric analysis). A receiver operating characteristics (ROC) curve analysis was performed to determine the optimal cutoff value of the D-index and c-D-index to predict the IFIs |
| Bias | 9 | Describe any efforts to address potential sources of bias | 4 | All clinical characteristics were collected by the retrospective chart review with de-identified patients' data, potentially bias from missing data. |
| Study size | 10 | Explain how the study size was arrived at | 7 | According to an original study of D-index predicted IFIs in various settings of AML patients with neutropenia [8], the D-index cutoff of 6,200 showed 100% sensitivity and 58% specificity along with the 15% prevalence of IFIs in AML patients in our center [17]. The sample size for adequate sensitivity was 100 patients with an acceptable margin of error of 5%. |

Continued on next page

| Quantitative variables | 11 | Explain how quantitative variables were handled in the analyses. If applicable, describe which groupings were chosen and why | 7 | The descriptive statistics were described as mean ± standard deviation (SD), median with interquartile range (IQR), or percentage values as appropriate. Dichotomous variables were compared using the Chi-square or Fisher's exact test. The test of normality was assessed by the Shapiro-Wilk test. Comparison of continuous variables between IFIs and no-IFIs group was analyzed by Student's *t*-test (for parametric analysis) or Mann-Whitney U test (for non-parametric analysis). A receiver operating characteristics (ROC) curve analysis was performed to determine the optimal cutoff value of the D-index and c-D-index to predict the IFIs |
| --- | --- | --- | --- | --- |
| Statistical methods | 12 | (*a*) Describe all statistical methods, including those used to control for confounding | 7 | as above in item no.11 |
|  |  | (*b*) Describe any methods used to examine subgroups and interactions | 7 | Dichotomous variables were compared using the Chi-square or Fisher's exact test. ... Comparison of continuous variables between IFIs and no-IFIs group was analyzed by Student's *t*-test (for parametric analysis) or Mann-Whitney U test (for non-parametric analysis). |
|  |  | (*c*) Explain how missing data were addressed | 7 | All patients with missing data were excluded. |
|  |  | (*d*) *Cohort study*—If applicable, explain how loss to follow-up was addressed  *Case-control study*—If applicable, explain how matching of cases and controls was addressed  *Cross-sectional study*—If applicable, describe analytical methods taking account of sampling strategy | - | - |
|  |  | (*e*) Describe any sensitivity analyses | 7 | Sensitivity, specificity, positive predictive value (PPV), and negative predictive value (NPV) were calculated using the cutoff values obtained from the ROC analysis. |
| Results | | | | |
| Participants | 13* | (a) Report numbers of individuals at each stage of study—eg numbers potentially eligible, examined for eligibility, confirmed eligible, included in the study, completing follow-up, and analysed | 8 | A total of 237 AML patients were recruited, 136 patients were excluded, and 101 AML patients who developed the first episode of febrile neutropenia after induction chemotherapy were subsequently enrolled, including 16 patients with IFIs and 85 AML patients without IFIs **(Figure 2)**. |
|  |  | (b) Give reasons for non-participation at each stage | Figure 2 | (All the details were provided in the figure 2.) |
|  |  | (c) Consider use of a flow diagram | Figure 2 | (All the details were provided in the figure 2.) |
| Descriptive data | 14* | (a) Give characteristics of study participants (eg demographic, clinical, social) and information on exposures and potential confounders | 8-9, Table 1 | The enrolled patients' characteristics were summarized **(Table 1)**. There were no significant differences in age, body mass index (BMI), serum albumin, the proportion of patients on sex, comorbidities, and cytogenetic risk of AML between the IFIs and no-IFIs groups.... |
|  |  | (b) Indicate number of participants with missing data for each variable of interest | Figure 2 | (All the details were provided in the figure 2.) |
|  |  | (c) *Cohort study*—Summarise follow-up time (eg, average and total amount) | 8 | Overall, the mean follow-up until ANC recovery was 22.6 ± 10.5 days. |
| Outcome data | 15* | *Cohort study*—Report numbers of outcome events or summary measures over time | 8 | 16 patients with IFIs and 85 AML patients without IFIs.  The mean duration of grade 4 neutropenia and profound neutropenia (ANC < 100 /μl) of patients with IFI were significantly longer compared with patients without IFIs (25.0 ± 12.1 days vs. 20.5 ± 5.3 days (*P* = 0.015) and 18.9 ± 7.9 days vs. 12.7 ±5.6 days (*P* < 0.001), respectively), as was the median D-index (8,803.3 (IQR: 7,746-9,923.8) vs. 4,312 (IQR: 2,311-6,274); *P* < 0.001). In addition, the median c-D-index of the IFI group was 6,535.5 (IQR: 5,498.5-8,944.5) compared with a total D-index of the no-IFI group of 4,312 (IQR: 2,311-6,274; *P* < 0.001). |
|  |  | *Case-control study—*Report numbers in each exposure category, or summary measures of exposure | *-* | *-* |
|  |  | *Cross-sectional study—*Report numbers of outcome events or summary measures | *-* | *-* |
| Main results | 16 | (*a*) Give unadjusted estimates and, if applicable, confounder-adjusted estimates and their precision (eg, 95% confidence interval). Make clear which confounders were adjusted for and why they were included | 8 | The mean duration of grade 4 neutropenia and profound neutropenia (ANC < 100 /μl) of patients with IFI were significantly longer compared with patients without IFIs (25.0 ± 12.1 days vs. 20.5 ± 5.3 days (*P* = 0.015) and 18.9 ± 7.9 days vs. 12.7 ±5.6 days (*P* < 0.001), respectively), as was the median D-index (8,803.3 (IQR: 7,746-9,923.8) vs. 4,312 (IQR: 2,311-6,274); *P* < 0.001). In addition, the median c-D-index of the IFI group was 6,535.5 (IQR: 5,498.5-8,944.5) compared with a total D-index of the no-IFI group of 4,312 (IQR: 2,311-6,274; *P* < 0.001). |
|  |  | (*b*) Report category boundaries when continuous variables were categorized | 8 | as the above |
|  |  | (*c*) If relevant, consider translating estimates of relative risk into absolute risk for a meaningful time period | - | - |

Continued on next page

| Other analyses | 17 | Report other analyses done—eg analyses of subgroups and interactions, and sensitivity analyses | 10  Figure 3A-D | ROC curve analysis of the D-index, with an area under the ROC curve (AuROC) of 0.937, showed better sensitivity and specificity to predict IFIs compared to the duration of grade 4 neutropenia with AuROC of 0.628 (*P* < 0.001).... |
| --- | --- | --- | --- | --- |
| Discussion | | | | |
| Key results | 18 | Summarise key results with reference to study objectives | 11 | This study demonstrated the performance of the D-index and c-D-index for evaluating neutropenic burden-associated IFIs in AML patients... |
| Limitations | 19 | Discuss limitations of the study, taking into account sources of potential bias or imprecision. Discuss both direction and magnitude of any potential bias | 13 | There were several limitations of this study... |
| Interpretation | 20 | Give a cautious overall interpretation of results considering objectives, limitations, multiplicity of analyses, results from similar studies, and other relevant evidence | 13-14 | Next, this study was entirely focused on AML patients who received induction remission and developed febrile neutropenia, resulting in limited in the generalizability. The different chemotherapy regimens in diverse disease populations could result in a different cutoff of the D-index in predicting IFIs or other infectious complications that needed further research. |
| Generalisability | 21 | Discuss the generalisability (external validity) of the study results | 13 | Next, this study was entirely focused on AML patients who received induction remission and developed febrile neutropenia, resulting in limited in the generalizability. |
| Other information | |  | | |
| Funding | 22 | Give the source of funding and the role of the funders for the present study and, if applicable, for the original study on which the present article is based | 14 | There was no funding in this study. |

*Give information separately for cases and controls in case-control studies and, if applicable, for exposed and unexposed groups in cohort and cross-sectional studies.

**Note:** An Explanation and Elaboration article discusses each checklist item and gives methodological background and published examples of transparent reporting. The STROBE checklist is best used in conjunction with this article (freely available on the Web sites of PLoS Medicine at http://www.plosmedicine.org/, Annals of Internal Medicine at http://www.annals.org/, and Epidemiology at http://www.epidem.com/). Information on the STROBE Initiative is available at www.strobe-statement.org.
