# Supplementary material for: D-index and invasive fungal infections (IFIs) in adult acute myeloid leukemia (AML) patients with the first episode of febrile neutropenia
Source: PLoS One. 2023 May 22;18(5):e0286089. doi: 10.1371/journal.pone.0286089 (PMC10202296; doi:10.1371/journal.pone.0286089)
Supplement: Attachment — Submitted filename: Response to reviewers.docx [file pone.0286089.s003.docx]

**Response to reviewers**

**Reviewer #1**

**1) Table 1. Please define the comorbidities in this study**

Response: I have added the details of comorbidities for both groups in the footnote of table 1, as suggested by the reviewer.

**2) Table 1. AML and IFI should be also spelled out in the Table 1.**

Response: I have already spelled out AML as "acute myeloid leukemia" and IFIs as "invasive fungal infections" in the footnote of table 1, as suggested by the reviewer.

**3) Table 1. What does "WBC per week" mean? In addition, please clarify its unit.**

Response: "WBC per week" referred to the WBC **"count"** per week, as specified in table 1. This phrase represented the frequency of serial blood cells counts per week. To provide a clearer and concise description, I have followed the reviewer's suggestion and added the unit of **"time"** after the term "WBC count per week".

**Reviewer #2**

**To facilitate the use of D-index and c-D-index please include the calculation spreadsheet in the supplementary material.**

Response: As suggested by the reviewer, I have provided the spreadsheet containing the calculation of the D-index in the supplement material or supporting information section.
